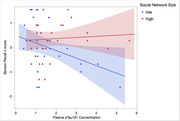# Social network size attenuates the negative effect of plasma pTau181 on memory and medial temporal lobe volumes

**DOI:** 10.1002/alz.090052

**Published:** 2025-01-09

**Authors:** Carina Lo, Lana S. Callies, Valentina E. Diaz, Coty Chen, Miwa Tucker, Savannah R. Hallgarth, Rowan Saloner, Shubir Dutt, Caitlin Wei‐Ming Watson, Kaitlin B. Casaletto, Joel H. Kramer, Emily W. Paolillo

**Affiliations:** ^1^ Memory and Aging Center, UCSF Weill Institute for Neurosciences, University of California, San Francisco, San Francisco, CA USA

## Abstract

**Background:**

Social health factors have been robustly associated with better cognitive health in older adults; however, less is known about how social network size affects the relationship between in‐vivo biomarkers of Alzheimer’s disease (AD) pathology and brain aging outcomes. We examined the independent and interactive relationships between plasma pTau181 and social network size on memory function and medial temporal lobe (MTL) volume in older adults.

**Method:**

Participants were 58 community‐dwelling older adults (mean age = 75.8 ± 5.6 years, mean education = 17.2 ± 1.8 years, 57% female, 91% cognitively unimpaired) enrolled in research at the UCSF Memory and Aging Center. Participants completed blood draws with plasma analyzed for pTau181 concentration, neuropsychological testing, brain MRI, and the Social Network Index, a self‐report questionnaire measuring a participant’s social network size (total number of people the participant contacts at least once every two weeks). To investigate whether social network size moderates the relationship between AD pathology and memory, multiple linear regression models examined the interactions between plasma pTau181 and social network size on verbal memory (California Verbal Learning Test–II delayed free recall), visual memory (Benson Figure recall), and bilateral MTL volume controlling for age, sex, education, and intracranial volume (MRI model).

**Result:**

Social network size and plasma pTau181 did not independently relate to MTL volume (*p*s>0.39), verbal memory (*p*s>0.53), or visual memory (*p*s>0.36). The interaction between social network size and plasma pTau181 on verbal memory was also not significant (*p* = 0.62). However, social network size significantly moderated the effect of plasma pTau181 on both visual memory (*β* = 0.33, 95%CI [0.13, 1.13], *p* = 0.015) and MTL volume (*β* = 0.27, 95%CI [0.05, 0.90], *p* = 0.03), such that negative effects of higher plasma pTau181 on worse visual memory and lower MTL volume were both attenuated with larger social network size.

**Conclusion:**

Consistent with prior literature, results suggest a potential neuroprotective effect of social network size on AD‐related cognitive and brain aging. These findings highlight the importance of promoting protective lifestyle factors, including social network size, to support brain health in those at risk for AD‐related cognitive decline.